# Probing local distortion around structural defects in half-Heusler thermoelectric NiZrSn alloy

**DOI:** 10.1038/s41598-020-76554-9

**Published:** 2020-11-13

**Authors:** Hidetoshi Miyazaki, Osman Murat Ozkendir, Selen Gunaydin, Kosuke Watanabe, Kazuo Soda, Yoichi Nishino

**Affiliations:** 1 Department of Physical Science and Engineering, Nagoya Institute of Technology, Nagoya, 466-8555 Japan; 2 Department of Natural and Mathematical Sciences, Faculty of Engineering, Tarsus University, 33400 Tarsus, Turkey; 3 School of Graduate Programs, Tarsus University, 33400 Tarsus, Turkey; 4 Department of Materials Physics, Nagoya University, Nagoya, 464-8603 Japan; 5 Synchrotron Radiation Research Center, Nagoya University, Furo-cho, Chikusa-ku, Nagoya, 464-8603 Japan; 6 Aichi Synchrotron Radiation Center, Aichi Science and Technology Foundation, 250-3 Minamiyamaguchi-cho, Seto, 489-0965 Japan

**Keywords:** Condensed-matter physics, Materials for energy and catalysis

## Abstract

The half-Heusler NiZrSn (NZS) alloy is particularly interesting owing to its excellent thermoelectric properties, mechanical strength, and oxidation resistance. However, the experimentally investigated thermal conductivity of half-Heusler NZS alloys shows discrepancies when compared to the theoretical predictions. This study investigates the crystal structure around atomic defects by comparing experimental and theoretical X-ray absorption fine structure (XAFS) spectra of the crystal structure of a half-Heusler NZS alloy. The results of both Zr and Ni *K*-edge XAFS spectra verified the existence of atomic defects at the vacancy sites distorting the *C*1_b_-type crystal structure. We concluded that the distortion of the atoms around the interstitial Ni disorder could be the probable reason for the observed lower thermal conductivity values compared to that predicted theoretically in half-Heusler alloys. Our study makes a significant contribution to the literature because the detailed investigation of the lattice distortion around atomic defects will pave the way to further reduce the thermal conductivity by controlling this distortion.

## Introduction

In the present scenario, significant reduction or ideally complete elimination of the heat losses—within mechanical and electrical devices, in general—are among the most important factors that are imperative for technological advancement. One of the foremost challenges of material-based research is to find sustainable methods of recycling the heat dissipated during a device’s usage. Thus, generating energy from the lost heat and again cooling the heated systems emerges as an ideal research area, and thermoelectric materials form the core of such technological research. In the current scientific studies, thermoelectric materials have attracted considerable attention of the researchers to recover the lost heat of energy production in every technological area where heat output is obtained. For this reason, scientists have extensively studied semiconductors to develop new and high-performance thermoelectric conversion materials that can work efficiently at both high and low temperature regimes^1,2^.

The dependency of thermoelectric efficiency on the physical properties of the material can be evaluated by the dimensionless formula:

$$zT = \sigma S^{2} T/\kappa ,$$

where the *σ* is the electrical conductivity, *S* is the Seebeck coefficient, *T* is the absolute temperature, and *κ* is the thermal conductivity. Further, the thermoelectric transformation can be increased by increasing the value of *zT*. Indeed, *κ* is the sum of the thermal conductivity contributed by the phonon and electrons, which can be inferred from the Wiedemann–Franz law^3^. The *C*1_b_-type half-Heusler Ni-based alloys with a band gap of approximately 0.3–0.4 eV have optimal amount of carriers (*n* ~ 10^19^/cm^3^) for thermoelectric conversion materials, possess a high Seebeck coefficient, and exhibit high electrical conductivity and thus, they can be effectively used as thermoelectric conversion materials at high temperatures around 600–1000 K^4–18^. Furthermore, in recent years, high thermoelectric properties (*zT* > 1.2) have been observed for both n-type and p-type materials by controlling their nanostructure precipitation, phase separation, and local crystal structure disorder, which resulted in a significant reduction in their thermal conductivity^13,16^. Thus, half-Heusler alloys are one of the most promising candidates for developing next generation thermoelectric conversion materials.

The half-Heusler NiZrSn (NZS) alloy is particularly interesting owing to its excellent thermoelectric properties, mechanical strength^19^, and oxidation resistance^20,21^, which has led to its commercialization for developing thermoelectric power generation devices that can operate in high-temperature environments. However, the experimentally investigated thermal conductivity of a half-Heusler NZS alloy shows discrepancies when compared to the available theories. The experimental thermal conductivity in the NZS alloy, which contributes to the high thermoelectric properties of the material, is approximately half of the theoretically calculated value that assumes a perfect crystal^22–25^ for the calculations. Further, this inconsistency has been hypothesized to be present owing to the existence of atomic defects at the vacancy sites^26–28^. The existence of the interstitial atomic defects at vacancy sites in the NZS alloy has been confirmed by the synchrotron radiation X-ray powder diffraction (SR-XRD) measurement technique and the results were analyzed by the Rietveld method^27^. Although the atomic coordination around the atomic defect is considered to be distorted, which may contribute to a significant reduction in the thermal conductivity, the lattice distortion has not been observed in the material. The SR-XRD technique is the best available method to investigate the periodic arrangement of atoms in the crystal structure; however, this technique fails to ascertain the local lattice distortions in the crystal, which were described above. In future, the possibility of investigating the detailed changes in the lattice distortion around the atomic defect may pave the way to further reduce thermal conductivity by controlling the amount of distortion. It will be a state-of-the-art development for engineering further high-performance thermoelectric conversion materials.

The most powerful experimental method for gaining information about the atomic coordination around a particular atom, such as an atomic defect, is the X-ray absorption fine structure spectroscopy (XAFS) technique using a tunable photon source, such as the synchrotron radiation. The element-selective XAFS data are treated into two parts: (1) X-ray absorption near edge structure or XANES (Near Edge XAFS) reflects electronic features in the spectrum that are strongly influenced by the type of nearest neighbor atoms, and (2) EXAFS (Extended XAFS) reflects the local periodicity of a wide range of neighboring atoms. Therefore, it is possible to obtain detailed information on the local crystal structure around atomic defects by comparing the experimental and theoretical XAFS spectra of the crystal structure. The theoretical framework assumes the lattice distortion to be a consequence of the atomic defects to perform ab initio band structure calculations. In this paper, we present the theoretical and experimental results of a half-Heusler NZS structural investigation, which include the lattice distortions produced by the atomic defects. These distortions were predicted by the ab initio band structure calculations and experimentally validated using the XAFS measurements.

## Results and discussion

**Figure 1:**
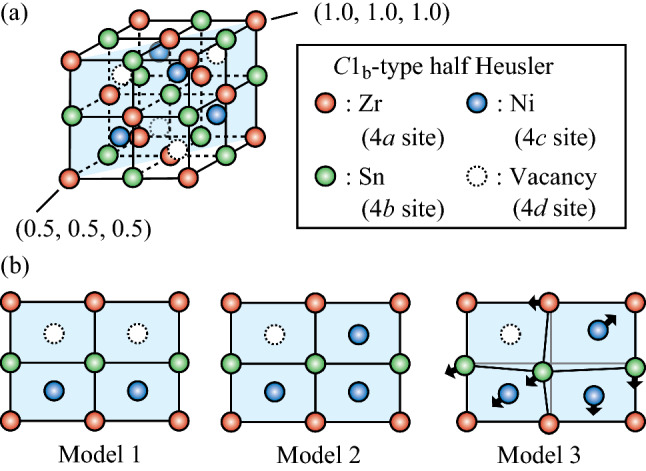
(**a**) Crystal structure of the C1_b_-type half-Heusler NiZrSn alloys. The shaded plane indicates the (110) mirror plane. The coordinates in the figure are the relative coordinates of Ni_36_Zr_32_Sn_32_ in the supercell. (**b**) Atomic coordinates of the various half-Heusler structures and their defect models: perfect crystal structure of the half-Heusler alloy (Model 1); an interstitial Ni disorder at one of the vacancy sites (Model 2); the atoms around the interstitial Ni disorder that are structurally relaxed to the optimal atomic position (Model 3). The arrows indicate the direction from the equilibrium position to the relaxed position.

Figure 1a, b show the model of the half-Heusler NZS crystal structure that was used for the band structure calculation. To calculate the effect of the interstitial Ni disorder at a vacancy site on the local crystal structure, the band structure calculations on the half-Heusler NZS alloy (Ni_36_Zr_32_Sn_32_) were performed. For calculation in the dilute interstitial Ni disordered system, 2 × 2 × 2 supercells for the half-Heusler alloy unit cell (Ni_4_Zr_4_Sn_4_) were used. The composition of the NZS alloy used in the XAFS measurements (Ni_34_Zr_33_Sn_33_) and the composition used in the band structure calculations were relatively consistent with each other. The details of the optimal atomic positions to which the coordinate positions of the constituent atoms structurally relax, and which are obtained by calculations, are given in the Supplemental Materials.

**Figure 2:**
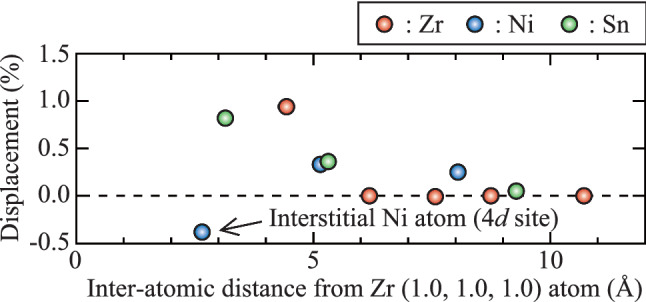
Displacement of the surrounding atoms of the (110) mirror plane from the equilibrium position, with respect to the Zr (1.0, 1.0, 1.0) atom.

To investigate the effect of the distortion of Ni interstitial defects on the surrounding atoms, optimal atomic positions were determined by structural optimization band calculations. Figure 2 shows the displacement of the surrounding atoms of the (110) mirror plane from the equilibrium position, with respect to the Zr (1.0, 1.0, 1.0) atom. The atoms around the interstitial Ni disorder are displaced from the equilibrium position, and the Zr atom next to the interstitial Ni disorder undergoes the highest displacement, which is about 0.9% of the equilibrium position. The displacement of the surrounding atoms from the equilibrium position decreases as their distance from the interstitial Ni disorder increases; further, a displacement of approximately 0.4% was observed even for the Sn atom, which was nearly 5.5 Å away from the interstitial Ni disorder. The interatomic distance of interstitial Ni atoms is sufficiently larger compare to the rage of influence of the local distortion. The local distortion effect has been properly calculated for the composition of Ni36Zr32Sn32 as assumed in the present calculations. Therefore, the existence of the interstitial Ni disorder resulted in the displacement of the lattice atoms from their equilibrium positions. This can explain the significant reduction in the experimental thermal conductivity of the NZS material when compared to its theoretically calculated thermal conductivity value.

**Figure 3:**
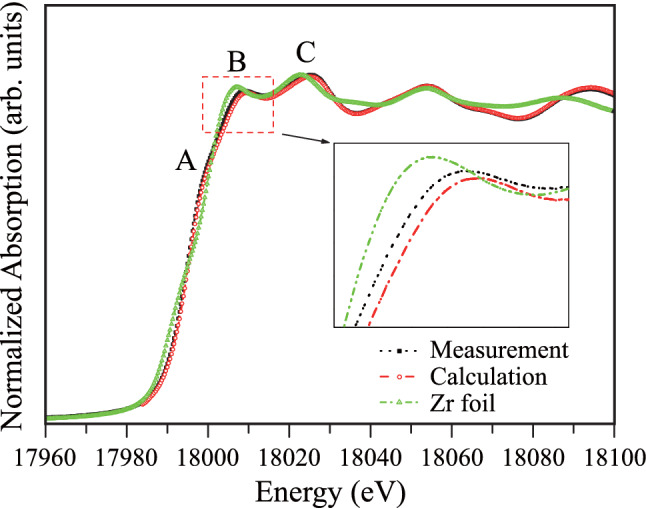
Experimental and calculated Zr *K*-edge XANES spectra of half-Heusler NiZrSn alloy. Zr *K*-edge XANES spectrum of Zr foil (reference sample), measured during the same beamtime, is also shown.

To evaluate the validity of the structural model with the ab initio band calculations that predict distortions due to the interstitial Ni disorder, the local crystal structure of the NZS alloy was experimentally investigated by the XAFS technique. The ground state electronic configuration of the Zr atom is [Kr] 4*d*^2^ 5*s*^2^; for the Zr^4+^ ionic state, the configuration becomes same as that of Kr, i.e., Zr^4+^ → [Kr] 4*d*^0^ 5*s*^0^ with empty *d* and *s* orbitals. The measured and theoretically calculated Zr *K*-edge XANES spectra of the half-Heusler NZS alloy are shown in Fig. 3 along with the Zr *K*-edge XANES spectrum of the Zr foil (reference spectrum). The peak features of both Zr metal and the half-Heusler NZS alloy agreed well with each other. This agreement highlights the similar crystal geometry and symmetries of the Zr atoms both in the Zr metal (cubic geometry with “Fm-3m”) and NZS alloy. The Zr *K*-edge absorption spectra of the NZS alloys showed typical characteristics; further, the shift of Zr^4+^ peak in the spectra was a result of the occurrence of the ionic state in the NZS alloy, which was different from the ground state. The Zr *K*-edge spectrum is a result of the excitation of the 1*s* electrons to the unoccupied 5*p* level as the final state. The empty 5*s* and 5*d* orbitals—lying below the 5*p* level—could provide convenient holes for the excited core electrons. However, owing to the quantum selection rules, *s → s* (1*s → *5*s*) and/or *s → d* (1*s → *4*d*) transitions are forbidden, *i.e.*, only *Δl* =  ± *1* transition is allowed. Therefore, the 1*s → *5*p* transition became the only route by which the 1*s* electrons could be excited to the higher lying levels. In Fig. 3, the Zr *K*-edge absorption spectra begin to rise at 17,970 eV and show a weak shoulder-like edge structure at 17,996 eV (for Zr-metal), assigned as “A,” and two main edge peaks, which were assigned as “B” and “C”. However, a shift of 2 eV towards the higher energy side was observed for the Zr *K*-edge spectra of the NZS material, which was a result of the ionic state of the Zr atoms in the NZS alloy.

The peak “A” at 17,998 eV for the NZS alloy occurs due to *d-p* mixing, which is also observed in the Zr *K*-edge spectrum^29^. The main peak corresponding to the 1*s → *5*p* transition shows typical split peaks at “B” (at 18,006.6 eV for Zr metal and 18,008.6 eV for NZS alloy) and “C” (at 18,022 eV for Zr metal and 18,024 eV for NZS alloy) for the NZS cubic geometry. The peak “B” is the main edge of the 1*s* to 5*p* transition of the Zr atoms consisting of tetrahedral site symmetry whereas the peak “C” is the main edge for the Zr atoms having octahedral site symmetry in the cubic NZS alloy. The electronic band gap was calculated as 0.395 eV with the FEFF 8.2 code, which is smaller than the band gap of 0.525 eV obtained from the band structure calculation of the NZS alloy without defects^30–33^. In this work, the calculated band gaps are smaller than those previously calculated. Such behavior can be explained by the fact that the band gaps shrink owing to structural distortion^34,35^.

The tail part of the XAFS spectra contains information regarding the environment of the source atom. The excited core electron of the source atom uses the excess energy as kinetic energy to travel among the neighboring atoms. The scattering of the photoelectrons creates fluctuations in the tail part of the XAFS spectra. The photoelectron scattering is a result of the impulse applied on the photoelectron by the outer shell electrons of the neighboring atoms. Consequently, the interference of the photoelectron wave vectors creates fluctuations in the EXAFS region of the spectra, which are positive when the interference is in phase and negative when the interference is out of phase. The information in the fluctuating region of the spectra could be processed by extracting the scattering parts using ATHENA and ARTEMIS programs. Apart from the electronic analysis of the structure, the effect of the molecular interplays could be observed on the arrangements of the neighboring atoms in the analysis of the EXAFS region. EXAFS part lies approximately 80 eV above the main absorption edge and within the energy range of 400–800 eV.

**Figure 4:**
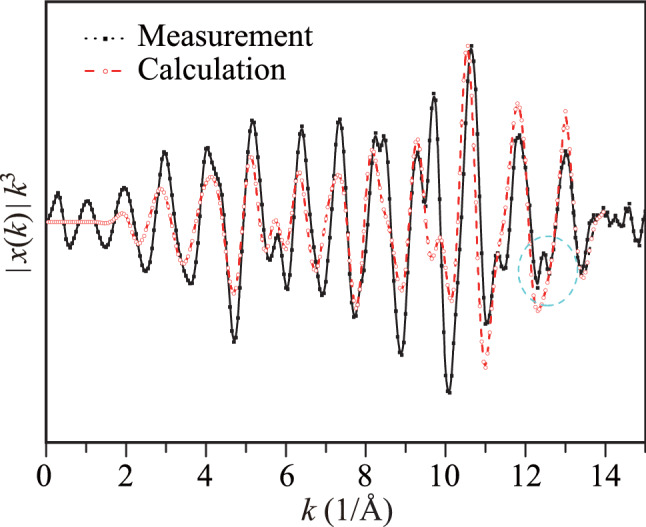
Experimentally measured and theoretically calculated Zr *K*-edge EXAFS scattering intensity of the half-Heusler NiZrSn alloy.

The EXAFS scattering intensity (*χ*) of the half-Heusler NZS alloy extracted from the Zr *K*-edge XAFS spectra is shown in Fig. 4 along with the corresponding theoretical calculations. We observed that the computational results were good elements to understand the complicated experimental results^29^. The third power of *χ* was obtained for the “*k*” range from 0 to 15 1/Å. The power of "*k*" is used as an indicator of atomic types in the scattering mechanism. A slight phase difference between the calculated and the measured spectra indicated that the crystal conditions in the real sample were significantly different from the assumed conditions, which were used in the theoretical calculations. A better agreement at the high *k* values would confirm harmony between the measured and calculated data. However, the noisy scattering data at the high *k* values indicated a disturbance in the vicinity of the source Zr atom, which was not observed in the theoretical calculations. Furthermore, an extra weak scattering intensity, shown by a dashed circle, implies a distortion in the first coordination shell of the NZS neighboring atom (*k* ~ 1/*λ*). Thus, Fourier transformation (FT) should be applied to the scattering data to obtain atomic distances and types in real space on a one-dimensional axis.

**Figure 5:**
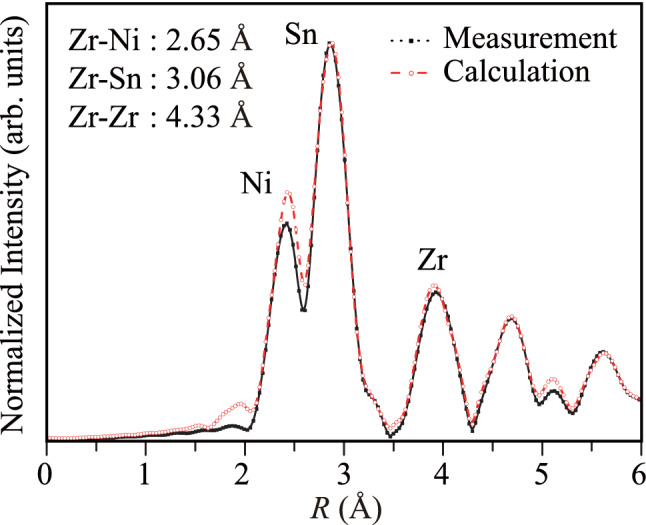
Experimentally measured and theoretically calculated Fourier transform of the Zr *K*-edge EXAFS scattering intensity comparison of half-Heusler NiZrSn alloy.

The FT of the scattering data is called "the radial distribution function" (RDF) that gives the atomic distances from the source atom (Zr), which are basically the atomic bond lengths in the crystal. The RDF data of the half-Heusler NZS alloy is given in Fig. 5. An analysis of the RDF data showed that the closest neighbor is the Ni atom coordination at a distance of 2.65 Å as the first-row neighbors. Beyond the Ni atoms, Sn atoms were determined at a distance of 3.06 Å, and the closest Zr atoms were determined at 4.33 Å.

**Figure 6:**
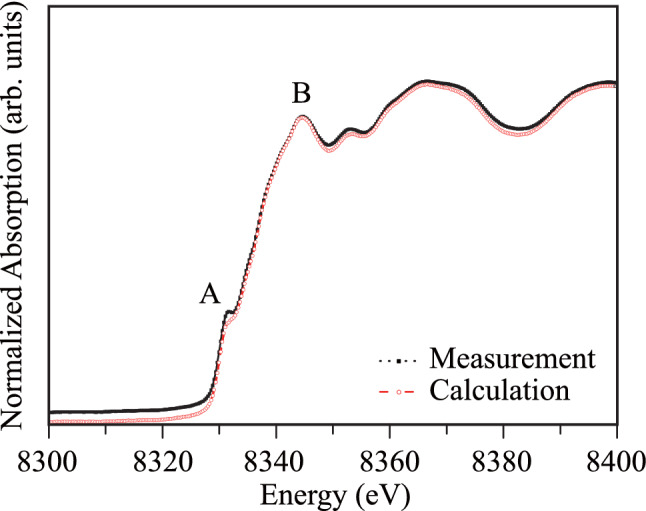
Experimentally measured and theoretically calculated Ni *K*-edge XANES spectra of the half-Heusler NiZrSn alloy.

The experimental and theoretically calculated *K*-edge XANES spectra of Ni atoms in the half-Heuser NZS alloy are presented in Fig. 6. The main K-absorption edge of Ni, denoted by “B” in Fig. 6, is a result of the 1*s → *4*p* transition; the electronic ground state of Ni is [Ar] 3*d*^8^4*s*^2^. The traces of distortion in the material showed that the row of the first neighboring atoms had crystal imperfections. Therefore, the next step of the study was to probe the Ni atoms in the half-Heuser NZS alloy. The final state for *s* electrons should not be a *d*-level or an *s*-level, according to the quantum selection rules. The Ni *K*-edge spectra begin to rise at 8319 eV and show a weak shoulder-like edge at 8331.3 eV, denoted by “A” in Fig. 6, which is due to the 1*s → *3*d* quadrupole transition that arises as a result of 3*d*-4*p* mixing of the Ni atomic levels. The main peak of the Ni *K*-edge has a maximum at 8344.6 eV, which was determined from the absorption characteristics of the Ni^3+^ ions in the NZS material. The theoretically calculated and the measured spectra show good agreement, and the weak peak feature beyond the peak “B” at 8352.9 eV is related to the multiple scattering of the photoelectron emitted from the source Ni atoms. The agreement between the measured data and the calculated data (performed with the generated data of the dilute interstitial Ni disorder system in NZS material) elucidate the crystal imperfections in the Ni atom row in the clusters of the measured sample.

**Figure 7:**
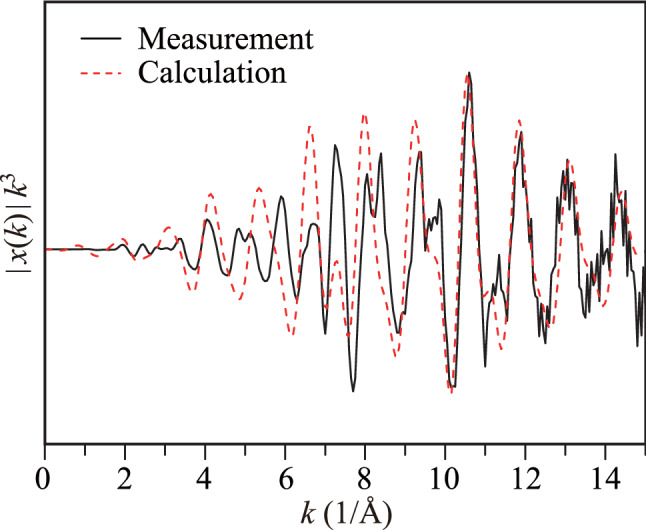
Experimentally measured and theoretically calculated Ni *K*-edge EXAFS scattering intensities of half-Heusler NZS alloy.

The electronic and structural properties of the Ni *K*-edge XANES spectra carry the traces of distortions in the Ni coordination of the NZS half-Heusler type alloy material. The EXAFS scattering data is the best tool to analyze this distortion in the crystal in detail. Figure 7 shows the *χ* scattering data of the photoelectron that was emitted from the source Ni atom. The scattering data of the photoelectron were harmonic in the high *k* region. However, the peak distortions in the high *k* region indicated crystal disorder at the Ni coordinations. A high kinetic energy photoelectron travels in an interstitial potential among the atoms. Therefore, any change in the atomic order or in the atomic type alters the behavior of the photoelectrons during their travel. Further, heavier atoms increase the interstitial potential leading to energy decay of the photoelectrons. The noise at the high *k* values emphasize the instability of the interstitial potential around the Ni atoms. This instability can provide important information regarding the crystal disorder in the Ni coordinations.

Recent direct observations of the band state and in the NZS compounds by means of photoelectron spectroscopy and detailed Hall's coefficient measurement suggest that structural defects cause a reduction in the mobility of the carriers and consequently an increase in the Seebeck coefficient^18,28^. Defect engineering has been identified as an effective strategy to improve the thermoelectric performance by controlling electron and phonon transport. Due to the presence of interstitial Ni atoms, the phonon is strongly scattered that results in reduced lattice thermal conductivity. The unique role of interstitials, if investigated in detail, can further encourage engineering thermoelectric materials by manipulating intrinsic disorder, especially in materials with complex structures.

## Conclusion

This study investigates the crystal structure around atomic defects by comparing experimental and theoretical XAFS spectra of the crystal structure of a half-Heusler NZS alloy. The XAFS measurements on half-Heusler NZS alloy showed that interstitial Ni disorder exists at the vacancy sites in the C1_b_-type crystal structure and the neighboring atoms around the atomic defects are distorted due to the existence of these defects. The observed lower experimental thermal conductivity, as compared to the theoretical thermal conductivity, in half-Heusler alloys is believed to be due to both the interstitial Ni disorder and the distortion effect around the defects. We observed that it is possible to control the atoms replacing the vacancy sites as well as the amount of interstitial atomic defects, which implies that there is a possibility of controlling the effect of distortion on the surrounding atoms as well. Therefore, we can conclude that the optimal defect control in the half-Heusler structure can further improve the scattering intensity and reduce the thermal conductivity in this material.

## Methods

The ab initio band structure calculations for perfect and disordered structures of the half-Heusler NZS alloys were performed using the *Vienna *ab initio* Simulation Package* (VASP) for the pseudopotential method^36–38^. In the calculation, the generalized gradient approximation (GGA) of Perdew, Burke, and Ernzerhof was used to express the exchange–correlation potential^39^, and the projector augmented wave (PAW) potentials were used for describing the valence and core electrons^40,41^. The first Brillouin zone was sampled with a 2 × 2 × 2 k-point mesh. The cut-off energy was set to 500 eV. The sampled mesh structure was optimized, iteratively, until the force on each atom became minimum. All calculations were performed using a large-scale parallel computer system, computer cluster NEC LX 1U server 110Rh-1, in the Research Center for Computational Science of Okazaki in Japan.

Stoichiometric NZS alloy was first prepared by arc melting of appropriate mixtures of 99.99% pure Ni and Zr, and 99.999% pure Sn, in an argon atmosphere. The ingot was crushed to a powdered form that contained particles with diameters less than 45 µm. To obtain a highly dense sample, the powder was consolidated to a disk by spark plasma sintering (SPS) at 1273 K under 50 MPa for 30 min in vacuum. The actual composition of the sintered NZS alloy was determined by X-ray fluorescence (XRF) spectroscopy and observed to be Ni_34_Zr_33_Sn_33_ (with an accuracy of 0.6), which indicated that the concentration of Ni in the sample was slightly more than that of Zn and Sn. For the XAFS measurements, powdered samples of dilute NZS with the boron nitride powder were prepared and pressed into pellets.

The Zr and Ni *K*-edges XAFS measurements, for the NZS alloy, were performed at the BL5S1 beamline of the Aichi Synchrotron Radiation Center. The incident X-ray beam was monochromatized by a Si (111) double-crystal monochromator. The measurements were performed at room temperature. The *K*-edge spectra for Zr and Ni were measured in the transmission mode using two ionization chambers, placed before and after the sample holder.

The analysis of the NZS alloy was supported by the XAFS calculations using real-space multiple scattering approach, as implemented in FEFF 8.2 code^42^. The calculations focused on the *K*-edges of the Ni and Zr atoms in the NZS alloy. For this purpose, input files for various half-Heusler NZS cluster models were generated by using the TkAtoms package, as a part of the IFEFFIT shell^43^. The lattice parameter of the NZS cluster was used as determined by experiments. The calculations were performed for a 12 Å thick, dilute and interstitial Ni disordered NZS material having cubic (*Fm-3m*) geometry. This system contained 407 atoms (Ni, Zr, and Sn). For the calculations, backscattering and phase shifts with single and multiple scattering paths were calculated to obtain the EXAFS spectra at the room temperature. The composition used in the calculation is Ni36Zr32Sn32, while the composition of the sample for which XAFS measurements were performed is Ni34Zr33Sn33. The Ni K-edge XAFS spectra will be the sum of the XAFS spectra from all the Ni atoms in the composition. In the experimental Ni34Zr32Sn32, the ratio of Ni atoms in the C1b half-Heusler structure, except for the interstitial Ni atom, is about 97%. On the other hand, the calculated composition of Ni36Zr32Sn32 is about 89%. The XAFS spectra are significantly affected by the Ni atoms in the C1b half-Heusler structure except for the Interstitial Ni atom, resulting in a Ni atom fraction of about 97% in the C1b half-Heusler structure. Hence, although the composition of NZS used in the calculations and experiments is different, the calculated and experimental results of XAFS spectra are comparable.

## Supplementary information

Supplementary Table S1.
