# Supplementary material for: Probing local distortion around structural defects in half-Heusler thermoelectric NiZrSn alloy
Source: Sci Rep. 2020 Nov 13;10:19820. doi: 10.1038/s41598-020-76554-9 (PMC7666166; doi:10.1038/s41598-020-76554-9)
Supplement: Supplementary file 1 — Supplementary Table S1. [file 41598_2020_76554_MOESM1_ESM.doc]

Supplemental Material

**Probing Local Distortion around Structural Defects in half-Heusler thermoelectric NiZrSn Alloy**

Hidetoshi Miyazaki^1*^, Osman Murat Ozkendir^2,3†^, Selen Gunaydin^3^, Kosuke Watanabe^1^, Kazuo Soda^4, 5, 6^, Yoichi Nishino^1^

*1. Nagoya Institute of Technology, Dept. of Physical Science and Engineering, 466-8555, Nagoya, Japan*

*2. Tarsus University, Institute of Natural Science, Dept. of Nanotechnology and Advanced Materials, 33400, Tarsus, Turkey*

*3. TarsusUniversity, School of Graduate Programs, 33400, Tarsus, Turkey*

*4. Nagoya University, Dept. of Materials Physics, 464-8603, Nagoya, Japan*

*5. Synchrotron Radiation Research Center, Nagoya University, Furo-cho, Chikusa-ku, Nagoya 464-8603, Japan*

*6. Aichi Synchrotron Radiation Center, Aichi Science and Technology Foundation, 250-3 Minamiyamaguchi-cho, Seto 489-0965, Japan*

^*^miyazaki@nitech.ac.jp

^†^ozkendir@tarsus.edu.tr

Table SI shows the contents of the CONTCAR file for interstitial Ni disordered half-Heusler NZS alloy (Ni_36_Zr_32_Sn_32_), that are structurally relaxed to the optimal atomic position.

Table S. I. Contents of the CONTCAR file for interstitial Ni disordered half-Heusler NZS alloy (Ni_36_Zr_32_Sn_32_), that are structurally relaxed to the optimal atomic position.

| Atomic species | Ni Zr Sn |  | 0.624 0.126 0.126 |
| --- | --- | --- | --- |
| Universal scaling | 1 |  | 0.376 0.126 0.874 |
| factor |  |  | 0.624 0.874 0.874 |
| Lattice vectors | 12.3666 0.000 0.000 |  | 0.376 0.874 0.126 |
|  | 0.000 12.3666 0.000 |  | 0.376 0.376 0.624 |
|  | 0.000 0.000 12.3666 |  | 0.624 0.624 0.624 |
| Number of aoms | 36 32 32 |  | 0.624 0.376 0.376 |
| Coordinate | Direct |  | 0.376 0.624 0.376 |
| Atomic coordinates | 0.128 0.128 0.128 |  | 0.876 0.876 0.876 |
| of Ni | 0.872 0.872 0.128 |  | 0.124 0.124 0.876 |
|  | 0.872 0.128 0.872 |  | 0.124 0.876 0.124 |
|  | 0.128 0.872 0.872 |  | 0.876 0.124 0.124 |
|  | 0.376 0.376 0.126 | Atomic coordinates | 0.000 0.000 0.000 |
|  | 0.624 0.624 0.126 | of Zr | 0.500 0.000 0.000 |
|  | 0.624 0.376 0.874 |  | 0.000 0.500 0.000 |
|  | 0.376 0.624 0.874 |  | 0.000 0.000 0.500 |
|  | 0.126 0.376 0.376 |  | 0.253 0.253 0.003 |
|  | 0.126 0.624 0.624 |  | 0.747 0.747 0.003 |
|  | 0.874 0.624 0.376 |  | 0.747 0.253 0.997 |
|  | 0.874 0.376 0.624 |  | 0.253 0.747 0.997 |
|  | 0.376 0.126 0.376 |  | 0.003 0.253 0.253 |
|  | 0.624 0.126 0.624 |  | 0.003 0.747 0.747 |
|  | 0.376 0.874 0.624 |  | 0.997 0.747 0.253 |
|  | 0.624 0.874 0.376 |  | 0.997 0.253 0.747 |
|  | 0.126 0.624 0.126 |  | 0.253 0.003 0.253 |
|  | 0.874 0.376 0.126 |  | 0.747 0.003 0.747 |
|  | 0.874 0.624 0.874 |  | 0.253 0.997 0.747 |
|  | 0.126 0.376 0.874 |  | 0.747 0.997 0.253 |
|  | 0.126 0.126 0.624 |  | 0.500 0.500 0.000 |
|  | 0.126 0.874 0.376 |  | 0.000 0.500 0.500 |
|  | 0.874 0.874 0.624 |  | 0.500 0.000 0.500 |
|  | 0.874 0.126 0.376 |  | 0.251 0.501 0.251 |
|  | 0.749 0.499 0.251 |  | 0.500 0.000 0.250 |
|  | 0.749 0.501 0.749 |  | 0.500 0.000 0.750 |
|  | 0.251 0.499 0.749 |  | 0.500 0.250 0.000 |
|  | 0.251 0.251 0.501 |  | 0.500 0.750 0.000 |
|  | 0.251 0.749 0.499 |  | 0.250 0.000 0.500 |
|  | 0.749 0.749 0.501 |  | 0.750 0.000 0.500 |
|  | 0.749 0.251 0.499 |  | 0.000 0.500 0.250 |
|  | 0.501 0.251 0.251 |  | 0.000 0.500 0.750 |
|  | 0.499 0.251 0.749 |  | 0.252 0.252 0.252 |
|  | 0.501 0.749 0.749 |  | 0.748 0.748 0.252 |
|  | 0.499 0.749 0.251 |  | 0.748 0.252 0.748 |
|  | 0.500 0.500 0.500 |  | 0.252 0.748 0.748 |
| Atomic coordinates | 0.254 0.000 0.000 |  | 0.251 0.749 0.251 |
| of Sn | 0.746 0.000 0.000 |  | 0.749 0.251 0.251 |
|  | 0.000 0.254 0.000 |  | 0.749 0.749 0.749 |
|  | 0.000 0.746 0.000 |  | 0.251 0.251 0.749 |
|  | 0.000 0.000 0.254 |  | 0.500 0.500 0.251 |
|  | 0.000 0.000 0.746 |  | 0.500 0.500 0.749 |
|  | 0.250 0.500 0.000 |  | 0.251 0.500 0.500 |
|  | 0.750 0.500 0.000 |  | 0.749 0.500 0.500 |
|  | 0.000 0.250 0.500 |  | 0.500 0.251 0.500 |
|  | 0.000 0.750 0.500 |  | 0.500 0.749 0.500 |
